# Gender Matters: Nonlinear Relationships Between Heart Rate Variability and Depression and Positive Affect

**DOI:** 10.3389/fnins.2021.612566

**Published:** 2021-05-13

**Authors:** Derek P. Spangler, Emily J. Dunn, Amelia Aldao, Nicole R. Feeling, Matthew L. Free, Brandon L. Gillie, Michael W. Vasey, DeWayne P. Williams, Julian Koenig, Julian F. Thayer

**Affiliations:** ^1^Department of Biobehavioral Health, The Pennsylvania State University, University Park, PA, United States; ^2^Department of Psychology, The Ohio State University, Columbus, OH, United States; ^3^Anxiety and Behavioral Health Services, Worthington, OH, United States; ^4^University of Pittsburgh Medical Center Sports Medicine Concussion Program, Department of Orthopedic Surgery, University of Pittsburgh, Pittsburgh, PA, United States; ^5^Department of Psychological Science, University of California, Irvine, Irvine, CA, United States; ^6^Section for Translational Psychobiology in Child and Adolescent Psychiatry, Department of Child and Adolescent Psychiatry, Heidelberg University, Heidelberg, Germany; ^7^University Hospital of Child and Adolescent Psychiatry and Psychotherapy, University of Bern, Bern, Switzerland

**Keywords:** heart rate variability, nonlinear, gender, emotion, depression, positive affect, autonomic nervous system, gender differences

## Abstract

Vagally mediated heart rate variability (vmHRV), a measure of the parasympathetic nervous system’s control over the heart, is often negatively related to maladaptive emotional outcomes. Recent work suggests that quadratic relationships involving these factors may be present; however, research has not investigated gender differences in these nonlinear functions. To address this gap, the current study tested for quadratic relationships between resting vmHRV and depression and positive affect while investigating gender differences in these relationships. Significant quadratic effects were found between resting vmHRV and reports of both depression symptoms and positive affect in women but not men. Specifically, the lowest levels of depression and the highest levels of positive affect were found at moderate vmHRV in women. These results suggest that examinations of vmHRV’s nonlinear associations require the consideration of gender. Our findings are interpreted based on proposed differential neuropsychological mechanisms of vmHRV in men versus women.

## Introduction

Heart rate variability (HRV), or beat-to-beat variation in heart rate, is a non-invasive metric of autonomic nervous system activity (Berntson et al., 1997). Although there are many HRV metrics with different physiological interpretations, vagally mediated heart rate variability (vmHRV) has received special attention as a biomarker of adaptive functioning (Allen et al., 2007; Porges, 2007b; Thayer and Lane, 2009). VmHRV refers to high-frequency (∼0.25 Hz) oscillations in heart rate that index the parasympathetic nervous system’s regulation of the heart via the vagus nerve (Saul, 1990; Malik et al., 1996). This is in contrast to metrics of overall HRV or low-frequency HRV which reflect a mixture of sympathetic and parasympathetic influences. The Neurovisceral Integration Model posits that the degree of resting vmHRV proxies the neural aspects of emotion regulation— that is, the degree of prefrontal cortex (PFC) inhibition over subcortical emotion circuits (Thayer and Lane, 2009; Thayer et al., 2012). Individuals with high relative to lower resting vmHRV are thought to exhibit greater regulation of maladaptive emotions. The Vagal Tank and Polyvagal Theories similarly posit that relatively higher resting vmHRV indexes greater psychophysiological reserves for self-regulation (Porges, 2007a; Laborde et al., 2018). Consistent with these perspectives, higher resting vmHRV has been associated with lower depression (Kemp et al., 2010) and higher positive affect (PA; Kok and Fredrickson, 2010).

### Nonlinear Relationships Between VmHRV and Emotion

Most research on this topic has characterized vmHRV’s relationships to emotion as strictly linear (for review, see Thayer and Lane, 2009). However, recent work has found support for a quadratic association between vmHRV and adaptive emotional outcomes in which moderate levels of vmHRV were associated with lower depression, higher life satisfaction (Kogan et al., 2013), and greater positive emotion (Kogan et al., 2014; Duarte and Pinto-Gouveia, 2017). In the same studies, participants with very low and very high levels of vmHRV reported less adaptive emotional outcomes (e.g., higher depression, less PA). These findings potentially shed light on a few studies reporting that vmHRV is *positively* (rather than negatively) related to maladaptive emotional outcomes such as depression (Rottenberg, 2007) and poor social competence (Eisenberg et al., 1995). Specifically, a positive and negative association involving vmHRV might exist within the same nonlinear function (Spangler and Friedman, 2017). Studies not testing quadratic terms may only characterize one linear piece of the larger nonlinear relationship between vmHRV and emotion.

One limitation of the research examining these quadratic functions is that it has not adequately considered gender: an important moderator of vmHRV (Koenig and Thayer, 2016) and its associations with brain activity (Nugent et al., 2011) and emotion (Verkuil et al., 2015). The quadratic vmHRV-emotion associations in prior studies (e.g., Kogan et al., 2013, 2014; Duarte and Pinto-Gouveia, 2017) might be unique to women. Consistently, some of these prior studies had either an exclusively female sample (Duarte and Pinto-Gouveia, 2017) or a mostly (64%) female sample (Kogan et al., 2014, Study 2). Other studies detecting similar nonlinear functions had samples with more balanced gender ratios. However, their samples were considerably older (Mean age = 40 in Kogan et al. (2014), Study 1 and Kogan et al., 2013)—corresponding to an age when gender differences in vmHRV decrease (Umetani et al., 1998). Whether or not nonlinear functions are unique to women requires clarification since prior studies did not examine gender as a moderator.

### Gender, VmHRV, and the Compensatory Hypothesis

Nonlinear relationships between vmHRV and depression/PA being limited to women has a basis in two evolutionary theories: *the tend-and-befriend theory and parental investment theory* (Trivers, 1972; Taylor et al., 2000). Both perspectives imply that the psychophysiological mechanisms of vagal responses differ between mammalian males and females. Below, we summarize both theories, which we then connect to the vmHRV literature in order to motivate our hypotheses regarding nonlinear functions and their gender differences.

According to the tend-and-befriend theory, female mammals exercise a larger role in caregiving relative to males and are therefore more likely to buffer themselves and their offspring from emotional distress (Taylor et al., 2000; Taylor and Master, 2011; Taylor, 2012). Therefore, in the face of high distress, women putatively exhibit heightened vagal activity and oxytocin reactivity—responses which are mounted to compensate for heightened distress and to promote affiliation. Males, in contrast, respond to distress with “fight-or-flight,” which involves both vagal and social withdrawal. Similarly, in the parental investment theory, women are more strongly influenced by selection pressures to care for offspring and exercise cautious mate selection (Bjorklund and Kipp, 1996; Bjorklund and Shackelford, 1999). These pressures promote greater attempts at emotion regulation and inhibitory control in the face of context-appropriate responses, thus promoting the delay of impulses for the benefit of offspring. Tend-and-befriend makes a direct connection between parasympathetic (vagal) function and compensatory responses, which is consistent with the Neurovisceral Integration Model where vmHRV proxies emotion regulation capacity (Thayer and Lane, 2009). Similarly, the emotion regulation and inhibitory control functions cited in parental investment theory bear considerable overlap to the neurobehavioral functions proxied by resting vmHRV in Neurovisceral Integration.

Taken together, both evolutionary theories imply that high vmHRV in women, relative to men, more strongly represents a compensatory response—i.e., greater emotion regulation efforts in response to higher maladaptive emotion. High vmHRV in men may instead represent lower maladaptive emotion achieved by tonic inhibition of subcortical threat circuits (consistent with Neurovisceral Integration; Thayer and Lane, 2009). Importantly, depression can be conceptualized as a threat-related response because: (1) rumination is a key feature of depression and involves perseverative thinking about past threats to the self (Nolen-Hoeksema, 2000), and (2) enhanced neural responses to social threat signal heightened risk for depression (Chan et al., 2009; Kujawa et al., 2015). If women’s high vmHRV indexes a compensatory response, then women should exhibit stronger positive relations of vmHRV to both threat-related responses (including depression) and emotion regulation. This is indeed consistent with prior evidence. First, higher vmHRV has been related to increased activity in the amygdala (a region implicated in threat processing) in women but decreased amygdala activity in men (Nugent et al., 2011). Second, relative to men, women exhibit stronger positive associations between vmHRV and emotion regulation ability (Williams et al., 2019). This compensatory response to threat and depressive symptoms has a neural basis in the PFC. According to Drevets et al. (2004, 2008), more severe depressive episodes are related to greater medial PFC activity (a region proxied by resting vmHRV; Thayer et al., 2012), and this heightened PFC activity is hypothesized to index a compensatory response to depressive episodes.

Evolutionarily based gender differences in vmHRV’s mechanisms may explain positive associations between vmHRV and depressive symptoms in females in contrast to consistently negative associations in males (Thayer et al., 1998; Verkuil et al., 2015; Jandackova et al., 2016; Jarczok et al., 2017, 2018). In these relationships, high vmHRV in women may represent an effortful compensatory emotion regulatory response (i.e., a “tend-and-befriend” response) to cope with greater depression (Thayer et al., 2003).

### Gender and Nonlinear Relationships Between vmHRV and Emotion

Positive associations may not fully characterize the broader relationship between vmHRV and depression in women. There are reports of negative associations between vmHRV and internalizing symptoms in women (Dishman et al., 2000; Henje Blom et al., 2010). The mixed associations between vmHRV and depression in women might be encapsulated by a larger nonlinear function that is not observed in men. If this is true, then the previously reported quadratic associations of vmHRV with depression and PA (a construct inversely related to depression and important for adaptive emotional function; Nutt et al., 2007) might be specific to women. Consistently, one study reported a significant negative association between vmHRV and depression in men and a null association in women with wider confidence limits, implying a nonlinear function in women (Jandackova et al., 2016).

Potential nonlinear vmHRV-emotion relationships in women but not men have grounding in evolutionary theory and previous studies. Gender differences are traditionally examined as average differences (e.g., greater depression in women than men overall; Feingold, 1994; Bianchin and Angrilli, 2012), However, evolved mechanisms more strongly affecting one gender (e.g., tend-and-befriend) are subject to phenotypic variation between persons (Archer, 2019). In this vein, a “tend-and-befriend” response to negative emotion (positive vmHRV-depression relationship) may represent only a subset of women for whom vagal reactions are pronounced in order to counteract high depression. In other women, there may be a more typical inverse relationship between vmHRV and depression-related measures, which may reflect fundamental links between very low vagal control and poor emotion regulation across genders (Thayer and Lane, 2009; Henje Blom et al., 2010).

In summary, the inverse relationship between vmHRV and depression may only appear in women from low to moderate vmHRV within a larger quadratic association. At higher levels of vmHRV (e.g., right side of function), greater vmHRV may represent a compensatory response to higher depression, emerging as a positive vmHRV-depression association. Supporting this possibility, clinical studies underscore exaggerated vagal activity as a compensatory response to stress which contributes to vasovagal syncope and emotional fainting—outcomes that are more common in women (van Lieshout et al., 1991; van Dijk and Sheldon, 2008; Alboni et al., 2021). In contrast to automatic emotion regulation strategies (e.g., Christou-Champi et al., 2015), we propose that the compensatory vagal response among women comprises more effortful regulation where interpersonal conflict is buffered via emotional and social labor (Hochschild, 1983; Wharton, 1999; Butler et al., 2006). Assuming the “tend-and-befriend” response does not influence men’s vagal function, men’s vmHRV-depression relationship should be strictly linear and negative. Since PA is inversely related to depression (Nutt et al., 2007), the aforementioned functions between vmHRV and PA might resemble those for depression except in the opposite direction.

Quadratic associations between vmHRV and emotion in women have grounding in Vagal Tank Theory (Laborde et al., 2018). In Vagal Tank Theory, relatively higher levels of vmHRV index greater psychophysiological resources (e.g., integration of neural, metabolic, and cognitive resources) that can be utilized for self-regulation and hence adaptive emotional responses. Through this lens, low vmHRV in women represents less self-regulatory resources which may lead to deficient emotion regulation and in turn higher depression and lower PA. Moderate vmHRV in women may represent greater self-regulatory resources that permit the context-appropriate emotion regulation and in turn less depression and higher PA. High vmHRV in women may represent even greater self-regulatory reserves that have been built up to deal with heightened depressive symptoms and low PA. Indeed, self-regulatory reserves—represented as emotion regulation capacity and resting vmHRV—can be built up over time through either increased exposure to stress or interventions targeting the integrity of stress-related neural pathways (e.g., physical exercise, mindfulness training, slow breathing) (Hansen et al., 2004; Gross et al., 2016; Wei et al., 2017; Hulbert and Anderson, 2018). Men’s strictly linear vmHRV-emotion associations may be due to their vmHRV similarly indexing the degree of self-regulatory resources (e.g., higher vmHRV, more emotion regulation) without the existence of a compensatory response at high vmHRV.

### Current Study

The current study explicitly examined gender differences in the linear and quadratic relationships between resting vmHRV and depression symptoms and PA. We aimed to clarify the work of Kogan et al. (2013, 2014) and Duarte and Pinto-Gouveia (2017) by testing whether the quadratic relations of resting vmHRV with depression and PA are limited to women. As noted above, we hypothesized that vmHRV in women (but not men) would show a U-shaped function with depression symptoms and an inverted-U function with PA. In men, we predicted that vmHRV would exhibit strictly linear negative and positive associations with depression and PA, respectively.

## Materials and Methods

### Participants

A total of 305 undergraduate students (58.69% female; mean age = 19.85, *SD* = 3.71; 70.82% Caucasian) participated in this study. After providing informed consent via a signed consent form, participants completed questionnaires. Participants were asked to abstain from alcohol, tobacco, caffeine, and vigorous exercise 6 h before the study. The university’s Institutional Review Board (Protocol #: 2014B0524) approved all study procedures, which were in accordance with the Declaration of Helsinki.

### Procedure

Upon entering the laboratory, participants were instructed on study procedures and then provided informed consent. Electrocardiography (ECG) leads were attached to disposable surface electrodes on the participant’s thorax. Participants next completed a 5-min (eyes-open) resting baseline where they were asked to remain as still and quiet as possible while ECG was continuously collected. After the resting baseline, participants completed a series of self-report questionnaires pertaining to emotion and self-regulation. Many of the questionnaires are of distal relevance to the primary study; we therefore only describe the questionnaires measuring depression symptoms and PA (see below). The order of the questionnaires was randomly counterbalanced across participants. Participants next completed a thought suppression task (Grisham and Williams, 2009). Since this task is unrelated to the aims of the current paper, its results are not reported here. It is unlikely that the thought suppression task influenced the current results since the task occurred after our focal metrics (vmHRV, depression and PA) were collected.

### Self-Report Measures

#### Depression

Depression was measured with the depression subscale of the Depression Anxiety Stress Scale (DASS-D), a valid and reliable measure of depression symptoms (Lovibond and Lovibond, 1995; in this sample: α = 0.95). The DASS-D consists of 14 items that are rated on a four-point (0–3) Likert scale^1^. Ratings were summed across items to yield an overall depression score (Lovibond and Lovibond, 1995). Given their skew, DASS-D scores were normalized with an inverse transformation of percentile ranked scores (Templeton, 2011).

#### Positive Affect

Trait PA was measured with the PA subscale of the Positive and Negative Affect Schedule (PANAS)—Trait form (Watson et al., 1988). The PANAS contains 20 items that were rated on a five-point (1–5) Likert scale, thus allowing participants to rate the extent to which they generally experienced 10 positive and 10 negative emotions. Ratings across the items were summed to index overall scores for positive and negative affect separately (Watson et al., 1988). The positive and negative affect subscales demonstrated good reliability: α = 0.89 and α = 0.88, respectively.

### VmHRV

Electrocardiography (ECG) was measured with three leads that were attached to adhesive spot electrodes on the thorax at a modified lead II configuration. ECG was digitally recorded (sample rate = 1,000 Hz) using the MindWare 2000D (MW2000D) Impedance Cardiograph package (MindWare Technologies, Ltd., Gahanna, OH). The ECG signal was processed in Mindware (HRV 2.51 Analysis software) in order to identify R-peaks and extract the interbeat interval (IBI) time series from the 5-min baseline. The IBI data were then entered into Kubios 2.0 software to calculate the vmHRV measures (Tarvainen et al., 2014). Prior to vmHRV estimation, IBI time series were detrended using smoothness priors in order to remove non-stationarities that could bias HRV estimates (Tarvainen et al., 2002). VmHRV was estimated across the entire 5-min baseline with the root mean square of successive differences (RMSSD), a well-established metric of vmHRV for both short-term (∼5 min) and long-term (∼24 h) recordings (Ali-Melkkila et al., 1991; Allen et al., 2007). In accord with standardized guidelines for vmHRV analysis, we natural logarithm transformed RMSSD in order to reduce its skew (denoted as lnRMSSD). VmHRV was also calculated as high-frequency HRV (HF-HRV). In further alignment with HRV analysis guidelines, HF-HRV was computed as mean absolute power (ms^2^) in the HF band (0.15–0.4 Hz) using autoregressive spectral analysis (model order = 16). HF-HRV was natural logarithm transformed in order to reduce its positive skew (denoted as lnHF-HRV). LnHF-HRV was highly correlated with lnRMSSD, r(303) = 0.96, *p* < 0.001, and results were identical when analyzing lnHF-HRV. Prior reports indicate that RMSSD is less influenced by respiration and is more reliable than HF-HRV (Penttilä et al., 2001; Kuss et al., 2008). We therefore employed lnRMSSD as our primary metric of vmHRV, and all substantive results are reported with lnRMSSD.

The autoregressive spectral analysis was also used to compute the peak frequency (Hz) of the spectral power in the HF (0.15–0.4 Hz) band. This metric has been identified as a cost-effective and accurate proxy for respiration rate (Thayer et al., 2002). The peak frequency of HF power (natural log transformed to reduce skew; denoted as lnHFpeak) was entered as a covariate in statistical models in order to rule out the potentially confounding influence of respiration rate on vmHRV (Grossman et al., 1991).

### Statistical Analysis

Linear and quadratic effects of vmHRV in men and women were tested with a multiple regression approach, in accord with Cohen et al. (2003). Emotion variables (depression symptoms and PA) were entered as dependent measures in separate regression models. Linear relationships between vmHRV and emotion variables (depression symptoms and PA) were tested with a linear term of lnRMSSD which was grand-mean-centered. Nonlinear relationships were tested with a quadratic term of lnRMSSD which was created by squaring mean-centered lnRMSSD.

The first regression model included both men and women and predicted depression symptoms (normalized DASS-D scores) with the: (1) linear term of RMSSD, (2) quadratic term of lnRMSSD, (3) lnHFpeak, (4) Gender (factorial: 1 = men; 2 = women), (5) lnRMSSD X gender, and (6) lnRMSSD^2^ X gender (Cohen et al., 2003). The regression model testing PA was also conducted across men and women and had the same predictors as above (1–6); however, it instead included PA as the dependent measure.

To more clearly test gender differences in vmHRV effects, we re-estimated regression models predicting depression and PA separately for men and women. The predictors entered into the gender-separated models were: (1) linear RMSSD, (2) quadratic RMSSD, and (3) lnHFpeak.

All regression effects were reported as standardized regression coefficients with 95% confidence intervals. In order to judge associations based on effect size, regression coefficients were also reported as partial correlation coefficients alongside with 95% confidence intervals. Since hypotheses were directional in nature, we report one-tailed *p*-values (alpha = 0.05) and one-tailed confidence intervals for all regression coefficients and correlations^1^. The regression coefficients and partial correlations yielded the same results (*p* < 0.05). Importantly, depression models were re-estimated after Winsorizing one outlier for the normalized DASS-Depression variable (from z = 3.5 to z = 3 and Winsoried again to z = 2.5). In both cases, findings of Winsorized analyses were identical to those in the Results (*p* < 0.05). Other variables indicated no outliers (z > |3|). Furthermore, we re-estimated all regression models with untransformed variables (e.g., raw RMSSD), and the results remained unchanged (*p* < 0.05).

## Results

**FIGURE 1:**
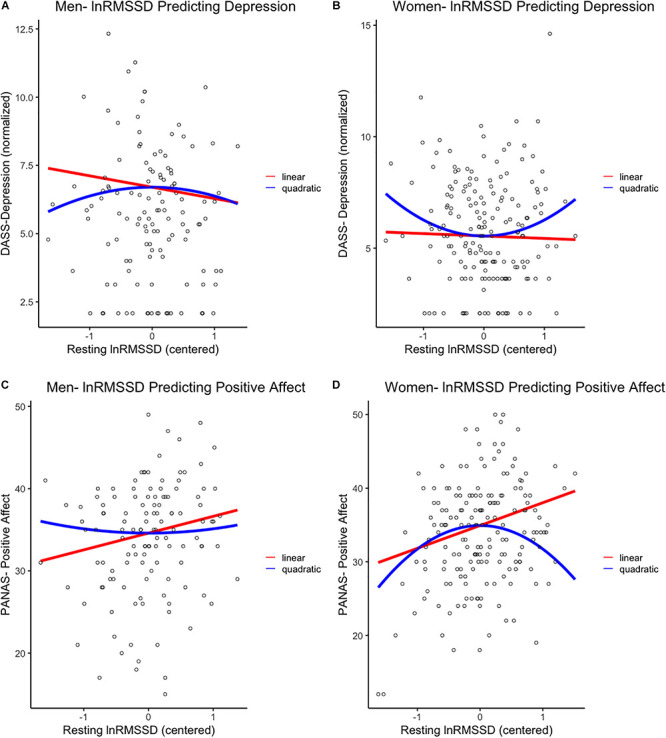
First row: Linear and quadratic effects of resting lnRMSSD on DASS-Depression scores in **(A)** men and **(B)** women separately. Second row: Linear and quadratic effects of resting lnRMSSD on PANAS-positive affect scores in **(C)** men and **(D)** women separately. LnRMSSD = natural log of root mean square of successive differences (in the natural log of millisecond units). LnRMSSD is grand-mean centered; DASS = depression anxiety stress scale. DASS-Depression scores are in inverse normalized units to reduce skew for parametric analyses. All results were identical (*p* < 0.05, one-tailed) when analyzing raw DASS-Depression scores. Significant (*p* < 0.05, one-tailed) quadratic effects were only detected in women, as depicted in **(B,D)**.

Table 1 contains descriptive statistics for study variables as well as independent samples *t*-tests examining gender differences in these variables. Our dependent measures—DASS-Depression scores and PA—were negatively correlated in the present sample, *Pearson product-moment r*(303) = −0.37, *p* < 0.001. Below, we report linear and nonlinear associations between vmHRV and emotion measures as a function of gender. These associations are depicted as partial correlations in Table 2 and plotted in Figure 1.

**TABLE 1:** Descriptive statistics.

	**Entire sample (*n* = 305)**		**Men (*n* = 125)**		**Women (*n* = 180)**		**Men vs. Women**	
	**Mean**	**SD**	**Mean**	**SD**	**Mean**		**t-test**	**Cohen’s *d* [95% CI]**
Ethnicity (% Caucasian)	71.1%	−	68.0%	−	73.3%	−	†	−
Age	19.85	3.71	20.23	3.61	19.59	3.77	1.48	0.17 [−0.06, 0.40]
**Cardiac measures**								
IBI	804.54	126.89	825.95	128.79	789.68	123.74	2.46*	0.29 [0.06, 0.52]
RMSSD	45.58	27.30	44.81	26.53	46.11	27.88	–0.41	−0.05 [−0.28, 0.18]
lnRMSSD	3.65	0.59	3.64	0.59	3.66	0.59	–0.42	−0.05 [−0.28, 0.18]
HF-HRV	1252.92	1443.86	1143.01	1316.60	1329.24	1524.94	–1.14	−0.13 [−0.36, 0.10]
lnHF-HRV	6.57	1.13	6.47	1.13	6.63	1.12	–1.23	−0.14 [−0.37, 0.09]
HFpeak	0.22	0.06	0.20	0.06	0.24	0.06	−5.31*	−0.62 [−0.85, −0.38]
LnHFpeak	−1.54	0.29	−1.65	0.28	−1.47	0.28	−5.43*	−0.63 [−0.87, −0.40]
**Self-report measures**								
DASS-D	8.79	9.84	8.72	10.06	8.83	9.71	–0.10	−0.01 [−0.24, 0.22]
DASS-D (normalized)	5.72	2.36	5.65	2.41	5.77	2.34	–0.44	−0.05 [−0.28, 0.18]
PANAS-PA	33.89	6.97	33.94	6.84	33.86	7.07	0.11	0.01 [−0.22, 0.24]
PANAS-NA	21.85	7.58	21.47	7.77	22.11	7.45	–0.72	−0.09 [−0.31, 0.14]

IBI, interbeat intervals (ms); RMSSD, root mean square of successive differences (ms); lnRMSSD, natural logarithm of RMSSD; HF-HRV, high-frequency heart rate variability; lnHF-HRV, natural logarithm of HF-HRV; DASS-D, Depression subscale of the Depression Anxiety Stress Scale; DASS-D (normalized), Depression scores in inverse normalized rank-order units with item substituted (see section “Materials and Methods”); PANAS-PA, Positive and Negative Affect Schedule- Positive Affect; PANAS-NA, Positive and Negative Affect Schedule- Negative Affect. Two-tailed *p < 0.05 for t-tests.

*T-tests that are not noted with an asterisk are not statistically significant (two-tailed p > 0.05). T-tests indicate that mean resting IBI was shorter and mean resting respiration rate was faster in women relative to men. However, effect sizes (Cohen’s d) suggest that compared to men, women had higher levels of vmHRV, depression, and negative affect, but lower levels of positive affect.*

*^†^Gender differences in ethnicity (proportion Caucasian) were tested with a chi-square test of independence. This test was not significant, χ^2^ = 1.02, two-tailed p = 0.312).*

**TABLE 2:** Partial Pearson correlation coefficients [95% CI] representing relationships between vmHRV and emotional outcomes.

	**DV: DASS-depression**			**DV: PANAS-positive affect**		
	**Entire sample**	**Men**	**Women**	**Entire sample**	**Men**	**Women**
LnRMSSD-linear	−0.05 [−0.14, 0.04]	−0.10 [−0.24, 0.05]	−0.03 [−0.15, 0.10]	0.03 [−0.06, 0.12]	0.17 [0.03, 0.31]*	0.26 [0.14, 0.37]*
LnRMSSD-quadratic	−0.08 [−0.17, 0.01]	−0.07 [−0.21, 0.08]	0.14 [0.02, 0.26]*	0.09 [−0.0007, 0.18]	0.04 [−0.11, 0.18]	−0.22 [−0.33, −0.10]*
Sex*LnRMSSD-linear	0.03 [−0.06, 0.13]	−	−	0.04 [−0.05, 0.13]	−	−
Sex*LnRMSSD-quadratic	0.10 [0.009, 0.19]*	−	−	−0.13 [−0.22, −0.04]	−	−

lnRMSSD, natural logarithm of the root mean square of successive differences (ms); DASS-D, Depression subscale of Depression Anxiety Stress Scale. Depression scores are in inverse normalized rank-order units with item substituted (see section “Materials and Methods”); PANAS-PA, Positive and Negative Affect Schedule- Positive Affect. LnRMSSD-linear represents the linear term of lnRMSSD. LnRMSSD-quadratic represents the square of the linear lnRMSSD term. Gender*LnRMSSD-linear represents the cross-product interaction between Gender (1 = men, 2 = women) and the linear lnRMSSD term. Gender*lnRMSSD-quadratic represents the product interaction between Gender (1 = men, 2 = women) and the quadratic lnRMSSD term.

*p < 0.05 based on one-tailed tests. All p-values are identical to those testing analogous regression coefficients (see section “Results”).

### Depression

In the model predicting depression across men and women, both the linear (β = −0.17, *p* = 0.200, 95% CI [−0.49, 0.16]) and quadratic terms (β = −0.27, *p* = 0.079, 95% CI [−0.59, 0.05]) were not significant. However, there was an interaction of gender with the quadratic (β = 0.35, *p* = 0.038, 95% CI [0.03, 0.67]) but not the linear term of lnRMSSD (β = 0.11, *p* = 0.285, 95% CI [−0.21, 0.43]). To better understand the interaction between the quadratic term of lnRMSSD and gender, we re-estimated the models predicting depression separately for men and women.

#### Men

In men, both the linear (β = −0.10, *p* = 0.135, 95% CI [−0.26, 0.05]) and quadratic (β = −0.07, *p* = 0.236, 95% CI [−0.22, 0.09]) effects of lnRMSSD on depression symptoms were not significant. These results indicate that there was no statistically significant relationship between vmHRV and depression in men. However, the negative direction of this linear effect is consistent with prior inverse linear relations between vmHRV and depressive symptoms in men but not women (e.g., Jandackova et al., 2016).

#### Women

In women, the quadratic (β = 0.14, *p* = 0.028, 95% CI [0.02, 0.27]) but not the linear (β = −0.03, *p* = 0.361, 95% CI [−0.15, 0.10]) effect of lnRMSSD on depression was statistically significant. These results indicate that, among women, the relationship between resting vmHRV and depression was quadratic rather than linear. Inspection of Figure 1 indicates that women’s quadratic relationship was *U-shaped* where moderate levels of lnRMSSD were associated with the lowest depression symptoms. Taken together, the shape of this nonlinear function resembles functions reported by Kogan et al. (2013) and was observed in women but not men.

### Positive Affect

In the model predicting PA across men and women, the linear (β = 0.09, *p* = 0.324, 95% CI [−0.23, 0.40]) and quadratic terms (β = 0.29, *p* = 0.059, 95% CI [−0.02, 0.60]) of lnRMSSD were not statistically significant. Importantly, there was a significant interaction of gender with the quadratic (β = −0.43, *p* = 0.012, 95% CI [−0.74, −0.11]) but not the linear term of lnRMSSD (β = 0.14, *p* = 0.223, 95% CI [−0.17, 0.46]). The latter results indicate that the quadratic relationship between lnRMSSD and PA varies by gender. We next stratified regression analyses by gender to better understand this interaction.

#### Men

Men evidenced a significant positive linear effect (β = 0.18, *p* = 0.028, 95% CI [0.03, 0.33]) but no significant quadratic effect of lnRMSSD (β = 0.04, *p* = 0.341, 95% CI [−0.11, 0.19]) on PA. These results indicate that, among men, higher resting vmHRV was significantly related to reports of higher PA, and this association was strictly linear.

#### Women

Women evidenced a positive linear effect of lnRMSSD (β = 0.26, *p* < 0.001, 95% CI [0.14, 0.38]) on PA, but this effect was qualified by a significant quadratic term (β = −0.21, *p* = 0.002, 95% CI [−0.33, −0.09]). These results indicate that relationship between vmHRV and PA in women can be characterized as quadratic as opposed to strictly linear. Consistent with this interpretation, Figure 1 revealed an inverted-U association between lnRMSSD and PA in women (but not men) where moderate levels of lnRMSSD were related to the highest levels of PA. Although only detected in women, this association is consistent with the functions of Kogan et al. (2014) and Duarte and Pinto-Gouveia (2017)^2^.

## Discussion

Consistent with Kogan et al. (2013), we found that resting vmHRV had a U-shaped quadratic relationship with depression symptoms where moderate levels of vmHRV were associated with the lowest levels of depression. Aligned with Kogan et al. (2014) and Duarte and Pinto-Gouveia (2017), we also found that vmHRV was quadratically related to trait PA, such that moderate vmHRV was associated with the highest levels of PA. As hypothesized, these quadratic relationships were detected in women but not men. In men, vmHRV appeared to exhibit strictly linear associations with depression and PA (the relation for depression was not significant but in the expected direction). Our findings highlight the importance of examining gender differences in vmHRV’s nonlinear associations with depression symptoms and PA. Depression involves a lack of PA, and, consistently, these constructs were negatively correlated in the current sample (Nutt et al., 2007). Therefore, our oppositely shaped quadratic relationship (inverted-U) between vmHRV and trait PA is a logical counterpoint to our depression findings in women. Taken together, these results suggest a broader relationship between vmHRV and adaptive emotional outcomes (Kogan et al., 2013). We speculate that prior research may have only detected linear relationships between vmHRV and emotional outcomes in women because they did not test for nonlinear associations.

### Gender Differences in VmHRV: Evolutionary Mechanisms

Extant theories of vagal function posit a strong link between resting vmHRV and emotion regulation capacity without directly positing a role of gender (Porges, 2007a, b; Thayer and Lane, 2009; Laborde et al., 2018). Expanding on these theories, the current study suggests that resting vmHRV in (some) women may tap into emotion regulation mechanisms that are different from those in men. Specifically, our findings are consistent with theorized gender differences in emotion regulation that arose from evolutionary selection pressures related to caregiving. The tend-and-befriend and parental investment theories posit that, in the face of high maladaptive emotion, women are more likely than men to mount an emotion regulatory response (e.g., effort to acquire social support, inhibit expression of emotional behavior) to divert resources to offspring (Bjorklund and Shackelford, 1999; Taylor et al., 2000). As suggested by tend-and-befriend, the compensatory response in women encompasses heighted parasympathetic activity (Taylor and Master, 2011). In this view, men are more likely to respond to maladaptive emotion with a prototypical fight-or-flight response involving vagal withdrawal and dis-inhibition of subcortical threat circuity.

The current linear associations between vmHRV and depression/PA in men (Figures 1A,C) reflect the “classic” mechanisms outlined in the Neurovisceral Integration Model, Vagal Tank Theory, and the “fight-or-flight” stress pattern. Here, relatively higher vmHRV reflects less threat responding achieved by automatic emotion regulation (i.e., tonic PFC-mediated inhibition of threat circuits) and, relatedly, greater self-regulatory resources (Thayer et al., 2012; Christou-Champi et al., 2015; Laborde et al., 2018). Critically, these strictly linear associations in males are consistent with prior reports (Carney et al., 1995; Thayer et al., 1998; Wang et al., 2013; Jarczok et al., 2017, 2018). In women, the same linear vmHRV-emotion relationships (positive for PA, negative for depression) were observed on the left side of the functions (Figures 1B,D). However, women with even higher levels of vmHRV (at the right side of the functions) exhibited reversal of these patterns, leading to: (i) a positive association between vmHRV and depression and (ii) a negative association between vmHRV and PA. These results are consistent with very high vmHRV representing a compensatory “tend-and-befriend” response to higher depression and lower PA (Thayer et al., 2003; Koenig and Thayer, 2016; Williams et al., 2019). In other terms, heightened parasympathetic function may reflect greater efforts to buffer maladaptive emotions that may otherwise impair tendencies related to caregiving and cautious mate selection (Bjorklund and Shackelford, 1999; Taylor et al., 2000; Taylor, 2012).

Very high levels of resting vmHRV at the right side of the function may also share key similarities with exaggerated vagal activity cited as a compensatory response in female vasovagal syncope patients (van Lieshout et al., 1991; Alboni et al., 2021). Furthermore, this greater compensatory effort at the right side of the function has been linked to augmentation in vmHRV among women (Butler et al., 2006). It may also encompass strenuous emotional labor that enhances women’s risk for emotional burnout and depression (Hochschild, 1983; Wharton, 1999). Consistent with these accounts, the present findings are aligned with Vagal Tank Theory (Laborde et al., 2018). Through this lens, moderate relative to low vmHRV in women indexes a greater degree of self-regulatory resources or strength. These greater resources are adaptive because they are critical to active self-regulatory efforts that, in turn, inhibit depressive symptoms and up-regulate PA (Joormann and Quinn, 2014). At high vmHRV (i.e., right side of function) in women, such increased vagal activation may index even greater self-regulatory resources that have been accrued to support emotion regulatory efforts aimed at counteracting high depression and low PA. Consistent with Vagal Tank’s view of vmHRV as an index of self-regulatory strength/reserves, resting vmHRV can be enhanced with training like a muscle. If this is true, then greater exposure to depression and low PA (for women with high vmHRV) may increasingly exercise emotion regulation circuits, in turn augmenting self-regulatory reserves and thus resting vmHRV (Troy and Mauss, 2011; Wei et al., 2017; Hulbert and Anderson, 2018). It should be noted that, like moderate vmHRV, high vmHRV in women may also represent an adaptive psychophysiological pattern that supports emotion regulation efforts. The particular emotion regulation mechanisms underlying very high vmHRV in women, although consistent with literature, are speculative and demand further investigation.

### Gender Differences and Potential Brain Mechanisms

The present findings might also reveal important central nervous system dynamics underlying gender differences in vmHRV and emotion regulation. For the entire functions in men and for the left side of the functions in women, relatively higher vmHRV may reflect greater tonic inhibition over subcortical circuits. In Neurovisceral Integration, such tonic inhibition is metabolically efficient, and it broadly supports context-appropriate emotional responses, expressed as less depression, higher PA, and other aspects of well-being (Thayer and Sternberg, 2006; Thayer and Lane, 2009; Geisler et al., 2010). At the right side of women’s nonlinear functions of women (i.e., positive vmHRV-depression relation), the neural mechanisms of higher vmHRV may represent PFC inhibition that is more metabolic costly and more phasic in nature (Jacobs and D’Esposito, 2011). Here, amygdalar and other subcortical responses are perhaps first elicited as default stress responses, and PFC-mediated inhibition is built up over time to compensate for such maladaptive subcortical activity. These possibilities are supported by evidence across disparate studies. Depressive episodes are correlated with increased medial PFC activity, perhaps to compensate for (or inhibit) heightened amygdalar activity (Drevets et al., 2004, 2008). Similarly, vmHRV is positively correlated with medial PFC activity, and women but not men evidence a positive association between vmHRV and amygdalar activity (Nugent et al., 2011; Thayer et al., 2012).

### Implications

The presence of nonlinear relationships in women but not men has important implications for the literature on vmHRV and emotion. First, the current results pose constraints on the quadratic vmHRV-emotion associations reported by Kogan et al. (2013, 2014) and Duarte and Pinto-Gouveia (2017). These authors claim that high resting vmHRV, generally speaking, should be re-evaluated as an adaptive biomarker and that it may even reflect aberrant regulatory processes. However, we show that this nonlinear relationship is not general but rather limited to women. By limiting the nonlinear function to women, evolutionary and psychophysiological theories of gender must be considered. Interpreting the nonlinear function through these perspectives casts very high vmHRV (in the nonlinear function) not as a maladaptive process *per se* but rather as an to attempt to compensate for high levels of depression, for example. We must also note that the effect sizes of our nonlinear relationships, like those in prior reports, are small. Therefore, the notion that a nonlinear function describes vmHRV’s relationships to any emotional variable must be cautiously interpreted and replicated in future research.

As a second implication, our nonlinear effects clarify previous conflicting findings in women. The direction of women’s vmHRV-depression associations is positive in some studies but negative in others (e.g., Thayer et al., 1998; Henje Blom et al., 2010). The current findings suggest that both the positive and negative relationships might exist within one nonlinear function. Third, the current nonlinear relations in women highlight how women and men differ in their neural regulation of specific maladaptive emotions: depression and low PA. These emotions are important because they are key risk factors in the etiology of clinical depression (Beaufort et al., 2017; de Jonge et al., 2017). Of course, depression and PA together cannot comprehensively assess maladaptive emotionality or other global constructs like adaptability. Anxiety and fear, which have also been related to vmHRV and psychopathological risk, could also elicit compensatory vagal reactions within a nonlinear function (Friedman, 2007). However, in the current study, there were no relationships (linear or nonlinear) between resting vmHRV and negative affect, suggesting some specificity in the emotional outcomes with which vmHRV is nonlinearly related.

### Limitations and Future Directions

As mentioned above, age is an important moderator of vmHRV and its relationships to gender. Our present sample was young (mean age = 19.85), so it is possible that the nonlinear relationships involving vmHRV would not replicate in an older sample. The magnitude of vmHRV and its gender differences have been shown to attenuate with increasing age (Umetani et al., 1998; Abhishekh et al., 2013). Similarly, both vagal and emotional function also appears to vary across the menstrual cycle, suggesting that present nonlinear relationships in women might be affected by female hormonal dynamics over time (Brar et al., 2015). Future studies should collect large samples with longitudinal designs in order to test this complex pattern of findings where vmHRV-emotion relationships and their gender differences vary across different timescales (e.g., menstrual cycle, age). The cross-sectional nature of our study also poses additional limitations. Notably, it is unclear whether the compensatory neural response represented by women’s high vmHRV in initiated concurrently or subsequently to heighted distress (e.g., higher depression). Longitudinal designs with experimental methods are required to elucidate: (i) the temporal dynamics and lead-lag relationships among emotional reactivity and regulation, and (ii) the casual effects of heighted emotional reactivity on precipitating regulatory/compensatory efforts.

As aforementioned, we only focused on depressive symptoms and PA as facets of maladaptive emotionality. It is hence unclear whether the present findings are specific to these emotional constructs or are more general. Evolutionary theories cited in the current paper largely mention coarse affective constructs like stress and emotion, thus pointing to a need to refine whether gender differences in emotion are general or domain-specific (Bjorklund and Kipp, 1996; Taylor et al., 2000). Future research needs to further explore vmHRV’s nonlinear relationships with other affective constructs.

## Conclusion

Despite limitations, we provide evidence that the nonlinear relations of vmHRV to depression and PA are limited to women. Within the nonlinear functions among women, very high vmHRV was related to greater depression and lower positive affect. These findings are consistent with exaggerated vmHRV indexing emotion regulatory efforts to cope with maladaptive emotionality. Taken together, the present findings further our understanding of the complex roles of vmHRV and gender in adaptive emotional outcomes.

## Data Availability Statement

The raw data supporting the conclusions of this article will be made available by the authors, without undue reservation.

## Ethics Statement

The studies involving human participants were reviewed and approved by the Ohio State University IRB. The patients/participants provided their written informed consent to participate in this study.

## Author Contributions

DS finalized analyses and wrote the manuscript with the assistance of JT. JK, DW, NF, MF, MV, and BG had a hand in designing the study and/or collecting and pre-processing the data. ED and AA assisted with analysis. All authors contributed to the article and approved the submitted version.

## Conflict of Interest

The authors declare that the research was conducted in the absence of any commercial or financial relationships that could be construed as a potential conflict of interest.

## Footnotes

1 Due to a technical error in collating the questionnaire materials, one item was missing from the DASS-D: “I felt that I had lost interest in just about everything.” To alleviate this issue, we substituted the missing DASS-D item with a highly similar item from the Post-Traumatic Stress Disorder (PTSD) Checklist- Civilian Version (PCL-C; Weathers et al., 1994): “Loss of interest in things that you used to enjoy?” In order to place items on a common scale, scores were divided by the maximum possible score and then summed to compute an overall depression score. The depression findings presented in the paper include the aforementioned substitution. Results were identical when the DASS-D item was omitted (*p* < 0.05).

2 Separate models were conducted with PANAS-negative affect as the dependent measure. None of the linear or quadratic effects of lnRMSSD were significant in the entire sample, or when examining men and women separately (results not presented).
